# Dynamics of Photoinduced Energy Transfer in Fully and Partially Conjugated Polymers Bearing π-Extended Donor and Acceptor Monomers

**DOI:** 10.3389/fchem.2020.605403

**Published:** 2020-11-05

**Authors:** Youngseo Kim, Na Yeon Kwon, Su Hong Park, Min Ju Cho, Dong Hoon Choi, Sungnam Park

**Affiliations:** Department of Chemistry, Research Institute for Natural Sciences, Korea University, Seoul, South Korea

**Keywords:** π-extended donor monomer, π-extended acceptor monomer, fully conjugated polymer, partially conjugated polymer, photophysical property, time-resolved fluorescence, energy transfer

## Abstract

The photophysical properties of donor (**D**)-acceptor (**A**) polymers were studied by designing two types of polymers, **(D-σ-A)**_******n**_ and **(D-π-A)**_******n**_, with non-conjugated alkyl (*sp*^3^) and π-conjugated (*sp*^2^) linkers using π-extended donor and acceptor monomers that exhibit planar A-D-A structures. The non-conjugated alkyl linker provides structural flexibility to the **(D-σ-A)**_******n**_ polymers, while the π-conjugated linker retains the rigid structure of the **(D-π-A)**_******n**_ polymers. Photoinduced energy transfer occurs from the large donor to acceptor units in both polymers. However, the photoinduced energy transfer dynamics are found to be dependent on the conformation of the polymers, where the difference is dictated by the types of linkers between the donor and acceptor units. In solution, intramolecular energy transfer is relatively favorable for the **(D-σ-A)**_******n**_ polymers with flexible linkers that allow the donor and acceptor units to be proximally located in the polymers. On the other hand, intermolecular (or interchain) energy transfer is dominant in the two polymer films because the π-extended donor and acceptor units in polymers are closely packed. The structural flexibility of the linkers between the donor and acceptor repeating units in the polymers affects the efficiency of energy transfer between the donor and acceptor units and the overall photophysical properties of the polymers.

## Introduction

Photoinduced energy transfer or charge transfer has been extensively studied using dyad- or triad-type small molecules composed of donor and acceptor moieties (Kuss-Petermann et al., 2012; Wiebeler et al., 2017; Wang et al., 2019). Most studies on the photophysical properties of such molecules have been carried out using boron dipyrromethenes or porphyrin as the donor moiety (Duvanel et al., 2013; Villamaina et al., 2013; Badgurjar et al., 2016). In dyad systems, the donor and acceptor moieties are connected by different types of linkers. The photophysical properties of dyad systems are largely influenced by the linkers between the donor and acceptor moieties; that is, π-conjugated (*sp*^2^-type) and non-conjugated (or aliphatic, *sp*^3^-type) linkers. In the past, various donor (**D**)- acceptor (**A**)-type dyad systems have been designed and synthesized as electronic and optoelectronic materials, including organic photovoltaic cells, organic field effect transistors, and organic light-emitting diodes, and their photophysical properties have been studied (Wu and Brand, 1994; Scholes, 2003; Schwartz, 2003; Murphy et al., 2004; Jones and Bradshaw, 2019). Although the focus has been on small-molecule donors and fullerene receptor systems (Jose et al., 2009; Caprasecca and Mennucci, 2014), a few studies have been performed on the photophysical properties of conjugated polymer systems. For example, Feng et al. introduced a single active polymer by binding a small molecule that exhibits acceptor properties as a side chain moiety to the main chain conjugated polymer with donor properties (Feng et al., 2019; Li et al., 2019). They observed new optoelectronic properties in this polymer by enabling energy transfer or electron transfer in the electronically excited state.

Our group reported polymer solar cells with two different copolymers synthesized by connecting conjugated donor- and acceptor-based macromolecular units (Lee et al., 2017). In our previous study, donor- and acceptor-based macromolecular units were connected using *sp*^3^ and *sp*^2^ linkers, respectively, to investigate the performance of polymer solar cells. The performance of polymer solar cells was found to dependent on the linkers between donor- and acceptor-based macromolecular units in the polymers. However, photoinduced energy transfer and photoinduced electron transfer in the two polymers were not able to be studied. Specifically, the donor- and acceptor-based macromolecular units were characterized by a large molecular weight distribution, making it difficult to obtain well-defined spectroscopic features in the UV-visible absorption and steady-state emission spectra. Generally, the photoinduced energy transfer and photoinduced electron transfer process in the polymers are important at the molecular level for optoelectronic devices and need to be comprehensively studied. The photoinduced energy transfer and photoinduced electron transfer in the polymers can be reliably studied by using the polymers with donor- and acceptor-based macromolecular units having a uniform molecular weight.

In this study, we designed and synthesized large A-D-A type donor monomers (**B3TP**, **D**) and A-D1-D2-D1-A type acceptor monomers (**B2IC**, **A**), and utilized them to synthesize two different polymers with non-conjugated alkyl linkers, **(D-σ-A)**_******n**_, and π-conjugated linkers, **(D-π-A)**_******n**_. The structural flexibility of **(D-σ-A)**_******n**_ and **(D-π-A)**_******n**_ is dictated by the linkers. Overall, **(D-σ-A)**_******n**_ is the partially conjugated polymer in which the π-extended donor and acceptor monomers are connected through flexible non-conjugated *sp*^3^ linkers. In contrast, **(D-π-A)**_******n**_ is the fully conjugated polymer in which the large donor and acceptor monomers are connected *via* conjugated *sp*^2^ linkers. It is very noteworthy that such polymer structures contain highly π-extended donor and acceptor monomers with a uniform molecular weight, and the large donor and acceptor monomers are alternatively arranged in the polymers. The photophysical properties of **(D-σ-A)**_******n**_ and **(D-π-A)**_******n**_ in toluene and films were investigated using UV-visible absorption, steady-state emission, and time-resolved fluorescence (TRF) spectroscopy, in conjunction with quantum chemical calculations. The photophysical properties of **(D-σ-A)**_******n**_ and **(D-π-A)**_******n**_ depend on the type of linker between the large donor and acceptor units in the polymers. The energy profiles of **(D-σ-A)**_******n**_ and **(D-π-A)**_******n**_ were found to be similar, but the photoinduced energy transfer from the donor to acceptor units occurred via different pathways. Photoinduced energy transfer in **(D-σ-A)**_******n**_ and **(D-π-A)**_******n**_ in toluene is mainly due to the intramolecular energy transfer, whereas photoinduced energy transfer in the **(D-σ-A)**_******n**_ and **(D-π-A)**_******n**_ films proceeds via both intramolecular and intermolecular (or interchain) energy transfer. This is the first report on the photophysical properties of partially and fully conjugated polymers containing well-defined π-extended donor and acceptor monomers. And our current results are expected to be applied to optoelectronic devices using π-conjugated donor-acceptor type polymers.

## Experimental

### Synthesis

#### Compound (D-σ-A)_n_

In a Schlenk tube, **M1** (85.3 mg, 28.8 μmol), **M2** (75 mg, 28.8 μmol), Pd_2_(dba)_3_ (1.3 mg, 5 mol%), and P(*o*-tolyl)_3_ (1.75 mg, 10 mol%) were dissolved in toluene (4 mL). The degassed binary mixture was stirred at 180°C for 2 h and subjected to cross-coupling Stille reaction under optimized microwave irradiation. The synthesized crude polymers were purified by precipitation into methanol, followed by Soxhlet extraction to remove unreacted monomers, undesired byproducts, and low-molecular-weight materials, using acetone, hexane, and dichloromethane in succession. The polymer solution was precipitated from methanol, and the solid was filtered to obtain polymer **(D-σ-A)**_******n**_ as a black solid [*M*_n_ = 24.04 kDa, polydispersity index (PDI) = 2.4]. Elemental Anal. Calcd. for (C_324_H_352_N_6_O_2_S_22_)_n_: C: 76.82; H: 6.96; N: 1.66; S: 13.92. Found: C: 76.18; H: 6.83; N: 1.71; S:13.62.

#### Compound (D-π-A)_n_

In a Schlenk tube, **M1** (130 mg, 44.1 mmol), **M3** (100 mg, 44.1 mmol), and Pd(PPh_3_)_4_ (5.0 mg, 10 mol%) were dissolved in toluene (4 mL). The synthesis method and reaction conditions were the same as described for the microwave synthesis of **(D-σ-A)**_******n**_. **(D-π-A)**_******n**_ is a black solid (*M*_n_ = 10.92 kDa, PDI = 2.3). Elemental Anal. Calcd. for (C_304_H_324_N_6_O_2_S_20_)_n_: C: 76.14; H: 6.86; N: 1.78; S, 9.55. Found: C:75.87; H: 6.76; N: 1.83; S: 9.32.

The chemical structures of **(D-σ-A)**_******n**_ and **(D-π-A)**_******n**_ were verified by gel permeation chromatography, ^1^H nuclear magnetic resonance (NMR; Supplementary Figures 2, 3), and elemental analyses (EA). The *M*_n_ values of the intermediate **(D-σ-A)**_******n**_ block and the final **(D-π-A)**_******n**_ were 20.0 and 10.9 kg mol^−1^, respectively, and the corresponding PDI values were 2.4 and 2.3, respectively. Because the molecular weight of the repeating units consisting of the large monomers is over 5,000 g/mol, the molar ratio of the **(D-σ-A)**_******n**_ and **(D-π-A)**_**n**_ repeating units was determined to be 1:1 from NMR spectra and theoretical EA calculations.

## Results and Discussion

### Design, Synthesis, and Characterization

The acceptor-donor-acceptor (A-D-A) backbone architecture of donor and acceptor monomers has drawn particular interest because of the easily tuned energy levels of these species. The benzo[1,2-*b*:4,5-*b*]dithiophene (BDT) unit used as the main core of π-extended donor and acceptor monomers in this study has become one of the most widely used polymer backbones because of its rigid planar conjugated structure, which can enhance electron delocalization and promote cofacial π-π stacking interactions in the solid state (Wang et al., 2018).

**Figure 1:**
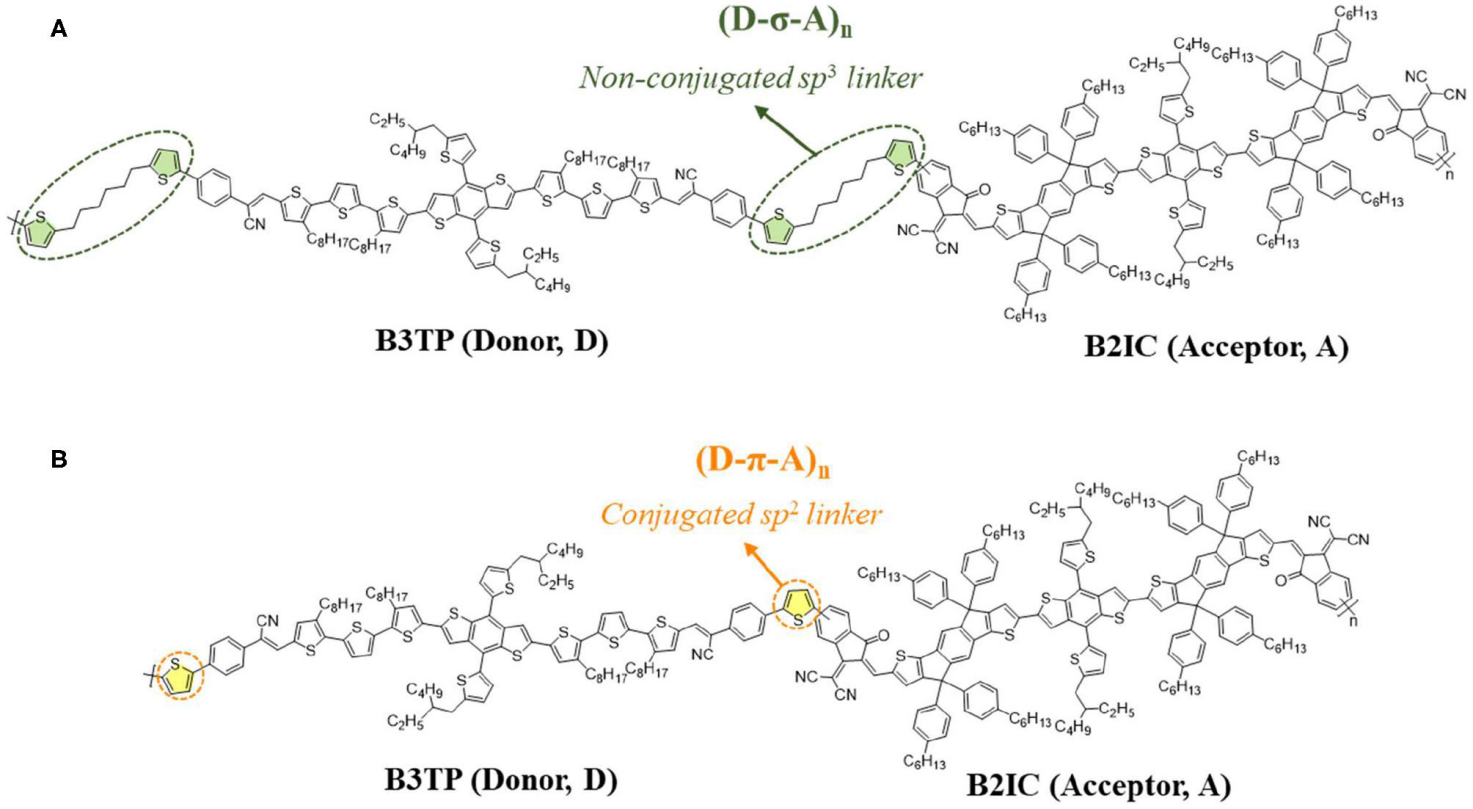
Molecular structures of **(A) (D-σ-A)**_******n**_ and **(B) (D-π-A)**_******n**_ polymers.

A design strategy for preparing effective A-D-A type donor monomers with narrow bandgaps involves increasing the electron donating ability of the donor monomers by extending the effective π conjugation length. The extended π-conjugated structure with three thiophenes increases the stiffness of the donor backbone. **B3TP**, as an A-D-A type conjugated donor monomer, was designed as shown in Figure 1. In **B3TP**, alkyl-substituted terthiophene moieties are added at both ends of the BDT core (Yang et al., 2018) and electron-withdrawing phenylacetonitrile groups are attached as the end-capped group, which guarantees a symmetric and planar structure. As an effective π-extended acceptor monomer, **B2IC** was designed in an acceptor-donor1-donor2-donor1-acceptor (A-D1-D2-D1-A) structure, as shown in Figure 1. Such A-D1-D2-D1-A type acceptors have rarely been reported. **B2IC** consists of the BDT core (D2) with indaceno[2,1-*b*:6,5-*b*]dithiophene moieties as an additional donor unit (D1) and the end-capping groups of 1,1-dicyanomethylene-3-indanone (IC) as an accepting unit (A).

In dyad systems, photoinduced energy transfer or photoinduced electron transfer is interesting photophysical phenomena that significantly depend on the linker type and length. To study such phenomena, we synthesized **(D-σ-A)**_******n**_ and **(D-π-A)**_******n**_ polymers by connecting the donor (**B3TP**) and acceptor (**B2IC**) units using an *sp*^2^ type conjugated linker (thiophene) and *sp*^3^ type non-conjugated linker. A detailed investigation of the photophysical properties is presented in the following sections.

### Theoretical Studies of π-Extended Monomers, (D-σ-A) and (D-π-A)

**Figure 2:**
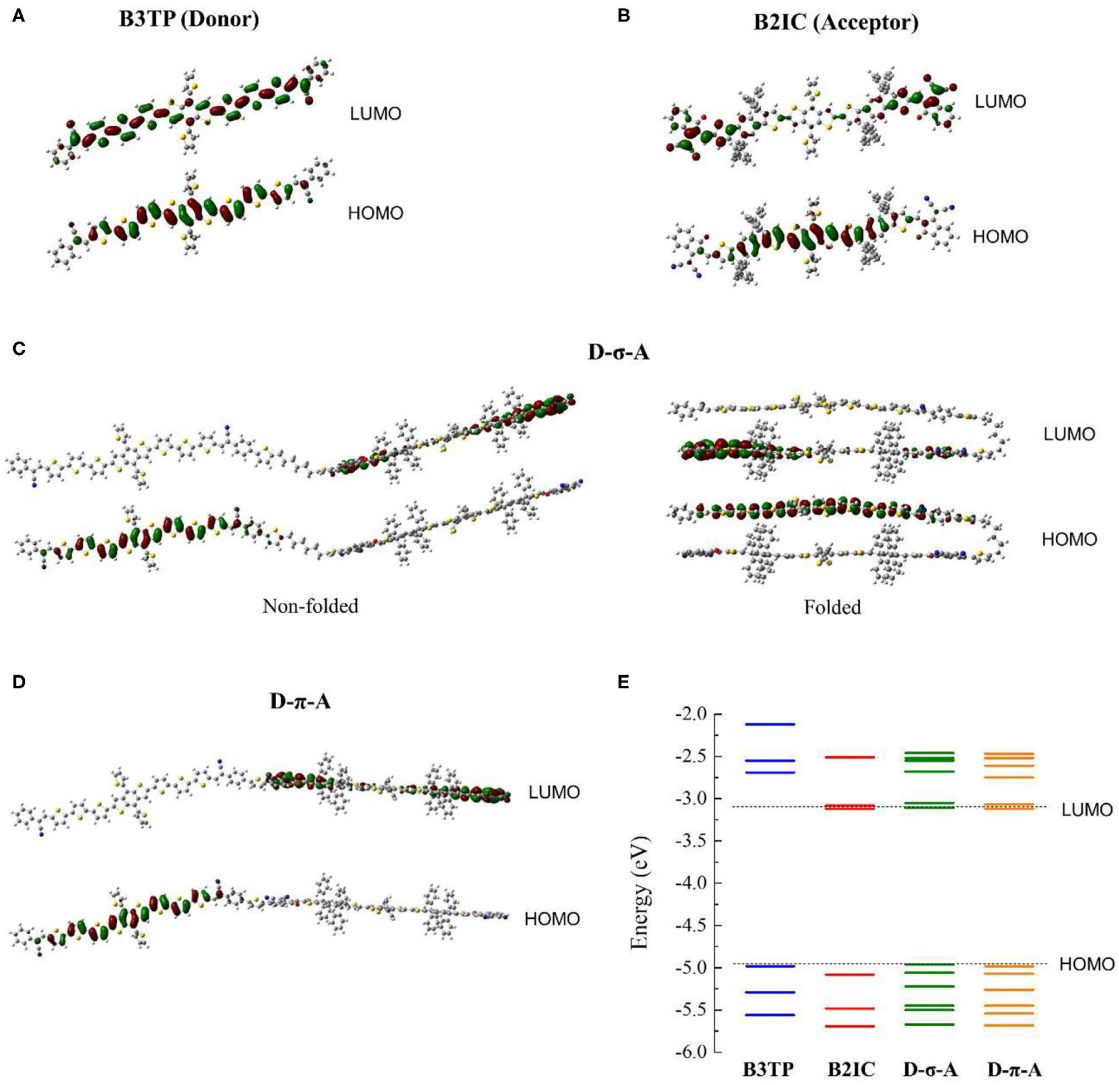
Optimized structures and frontier orbitals (HOMO and LUMO) of **(A) B3TP**, **(B) B2IC**, **(C) D-σ-A**, and **(D) D-π-A**. **(E)** Calculated electronic energies.

To study the electronic and optical properties, density functional theory (DFT) calculations [B3LYP/6-31g(d) and B3LYP-d3/6-31g(d)] were carried out. First, the optimized structures of **B3TP**, **B2IC**, **D-σ-A**, and **D-π-A** were obtained. The frontier orbitals (HOMO and LUMO) and natural transition orbitals (NTOs) were calculated. Figures 2A,B show the optimized structures and the HOMO and LUMO of **B3TP** and **B2IC**, respectively. The donor monomer, **B3TP** (A-D-A type), and the acceptor monomer, **B2IC** (A-D1-D2-D1-A type), are both planar and fully conjugated. Both **B3TP** and **B2IC** exhibit intramolecular charge transfer upon electronic transition (Supplementary Figure 4).

The optimized structures and frontier orbitals of **D-σ-A** and **D-π-A** are shown in Figure 2. Note that the folded structure of **D-σ-A** in Figure 2 is also energetically stable and can possibly be populated. Because **D-σ-A** and **D-π-A** have the same donor and acceptor units, but different linkers (*sp*^3^ vs. *sp*^2^) between the donor and acceptor units, the calculated electronic energies of the HOMO and LUMO levels are almost the same (Figure 2E). For both **D-σ-A** and **D-π-A**, the HOMO and LUMO are spatially localized at the π-extended donor and acceptor units, respectively, and exhibit strong charge transfer characteristics, which is directly associated with the photophysical properties of **(D-σ-A)**_******n**_ and **(D-π-A)**_******n**_, as discussed hereinafter.

### Spectroscopic Studies of π-Extended Monomers, (D-σ-A)_n_, and (D-π-A)_n_

**Figure 3:**
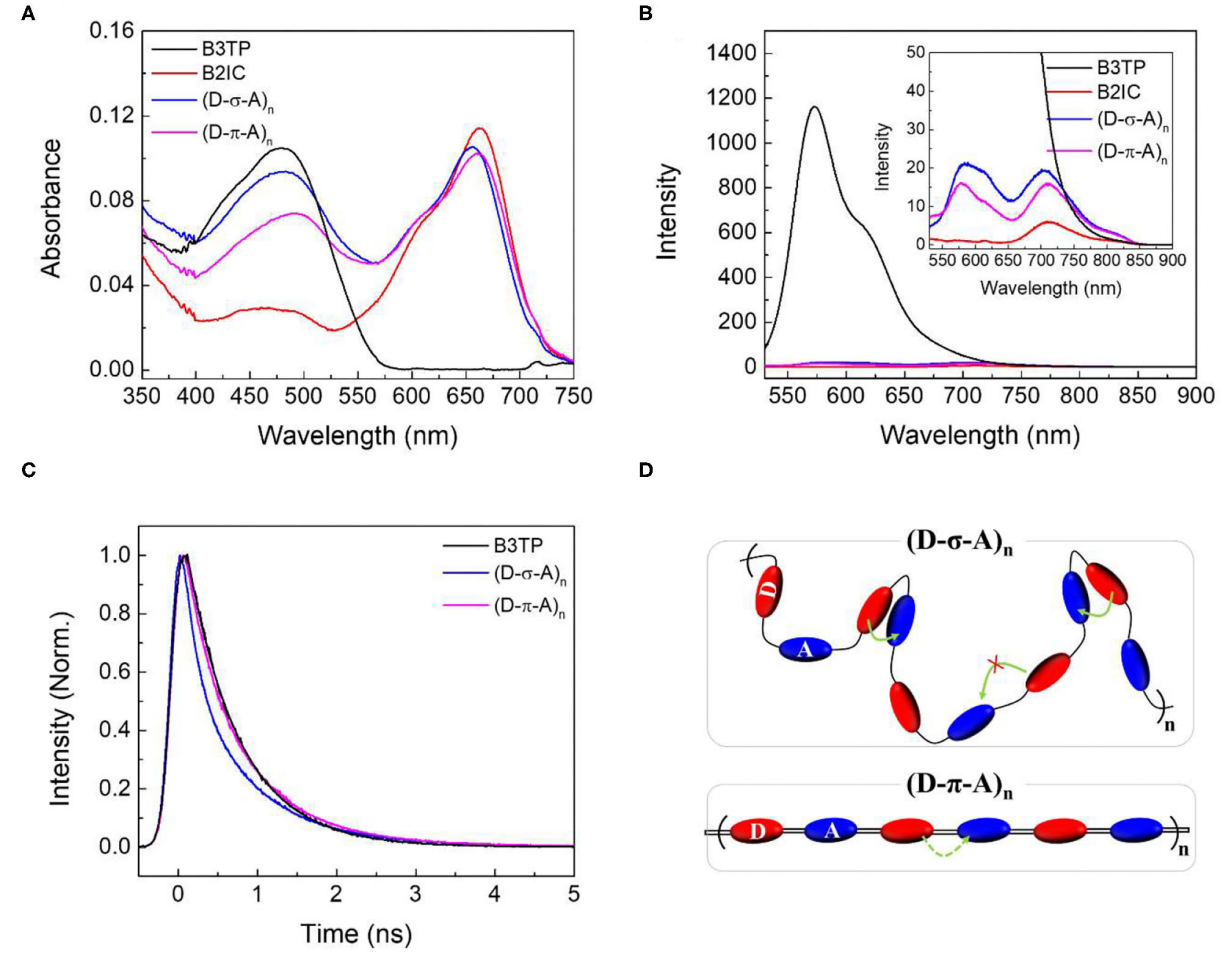
**(A)** UV-visible absorption spectra and **(B)** steady-state emission spectra of **B3TP**, **B2IC**, **(D-σ-A)**_******n**_, and **(D-π-A)**_******n**_ in toluene. Steady-state emission spectra were measured at the excitation of λ_ex_ = 520 nm. **(C)** Time-resolved fluorescence signals were obtained at 590 nm at the excitation of λ_ex_ = 520 nm. The concentration of **B3TP**, **B2IC**, **(D-σ-A)**_******n**_, and **(D-π-A)**_******n**_ in toluene is 0.2 μM. **(D)** Schematic illustration of **(D-σ-A)**_******n**_ and **(D-π-A)**_******n**_ in toluene and the energy transfer between π-extended donor and acceptor units. Intramolecular Förster resonance energy transfer in toluene is indicated by curved arrows.

The UV-visible absorption and steady-state emission spectra of **B3TP**, **B2IC**, **(D-σ-A)**_******n**_, and **(D-π-A)**_******n**_ in toluene are shown in Figure 3. The UV-visible absorption spectra of **(D-σ-A)**_******n**_ and **(D-π-A)**_******n**_ are similar, with two absorption peaks near 480 and 660 nm, representing the donor and acceptor units (**B3TP** and **B2IC**), respectively. The emission spectrum of **B3TP** (Figure 3B) exhibits an intense peak at 590 nm (λ_ex_ = 520 nm), whereas **B2IC** shows very weak emission at 710 nm. **B3TP** is nearly planar and structurally rigid, which seems to give a relatively large fluorescence quantum yield. In contrast, **B2IC** contains 8 phenyl groups in the donor (D1) units, which are flexible and freely rotatable in toluene and might be responsible for significant fluorescence quenching.

As shown in the inset of Figure 3B, **(D-σ-A)**_******n**_ and **(D-π-A)**_******n**_ exhibit two emission peaks at 590 and 710 nm (λ_ex_ = 520 nm), resulting from **B3TP** and **B2IC**, respectively. However, the overall fluorescence of **(D-σ-A)**_******n**_ and **(D-π-A)**_******n**_ is significantly quenched when compared with that of **B3TP**. This observation can be readily explained based on the fluorescence properties of **B3TP** and **B2IC** as follows. The donor units (**B3TP**) in **(D-σ-A)**_******n**_ and **(D-π-A)**_******n**_ are directly excited by 520 nm photons. The excited donor units in **(D-σ-A)**_******n**_ and **(D-π-A)**_******n**_ undergo Förster resonance energy transfer (FRET) to the acceptor units (**B2IC**) in **(D-σ-A)**_******n**_ and **(D-π-A)**_******n**_, owing to which, the fluorescence is quenched. FRET is readily possible in **(D-σ-A)**_******n**_ and **(D-π-A)**_******n**_ because the emission spectrum of the donor (**B3TP**) and UV-visible absorption spectrum of the acceptor (**B2IC**) overlap (Supplementary Figure 5), and the distance between the donor and acceptor units in the polymer backbones is sufficiently small.

**Figure 4:**
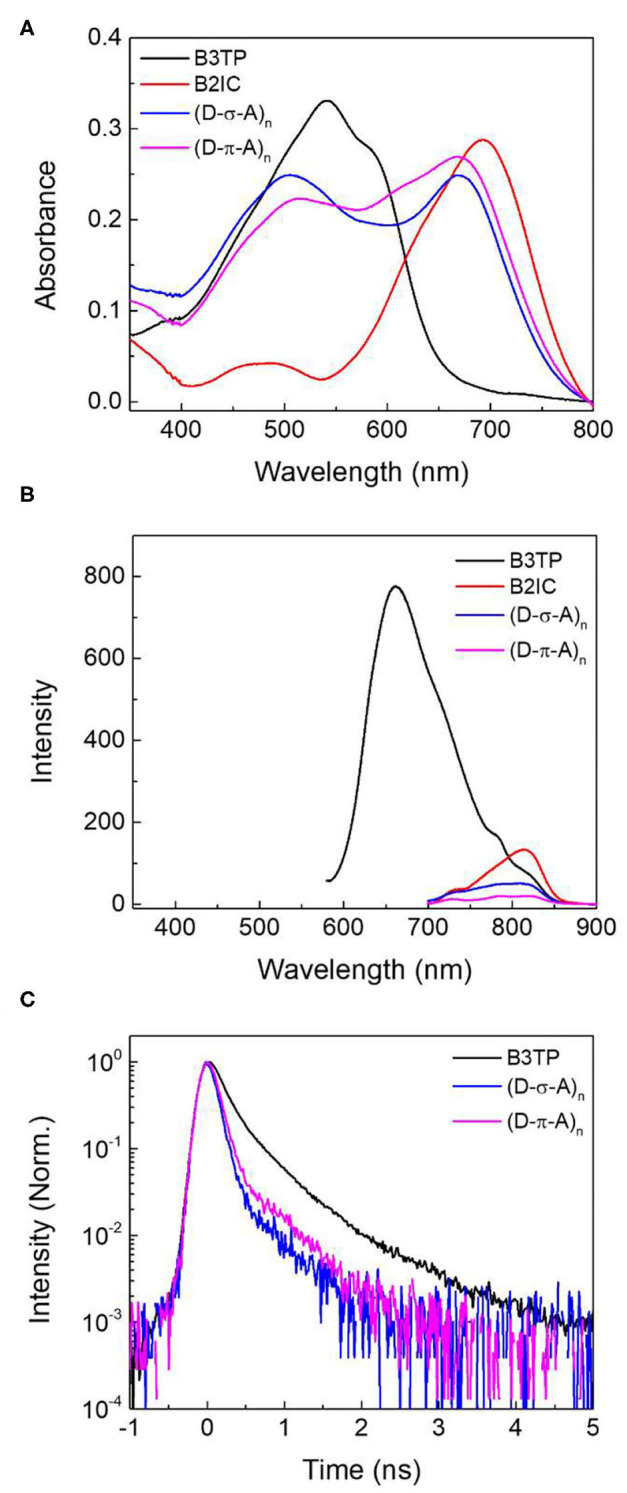
**(A)** UV-visible absorption spectra and **(B)** steady-state emission spectra of **B3TP**, **B2IC**, **(D-σ-A)**_******n**_, and **(D-π-A)**_******n**_ in films. The steady-state emission spectra were measured at the excitation of λ_ex_ = 520 nm. **(C)** Time-resolved fluorescence signals were obtained at 670 nm (λ_ex_ = 520 nm).

**Figure 5:**
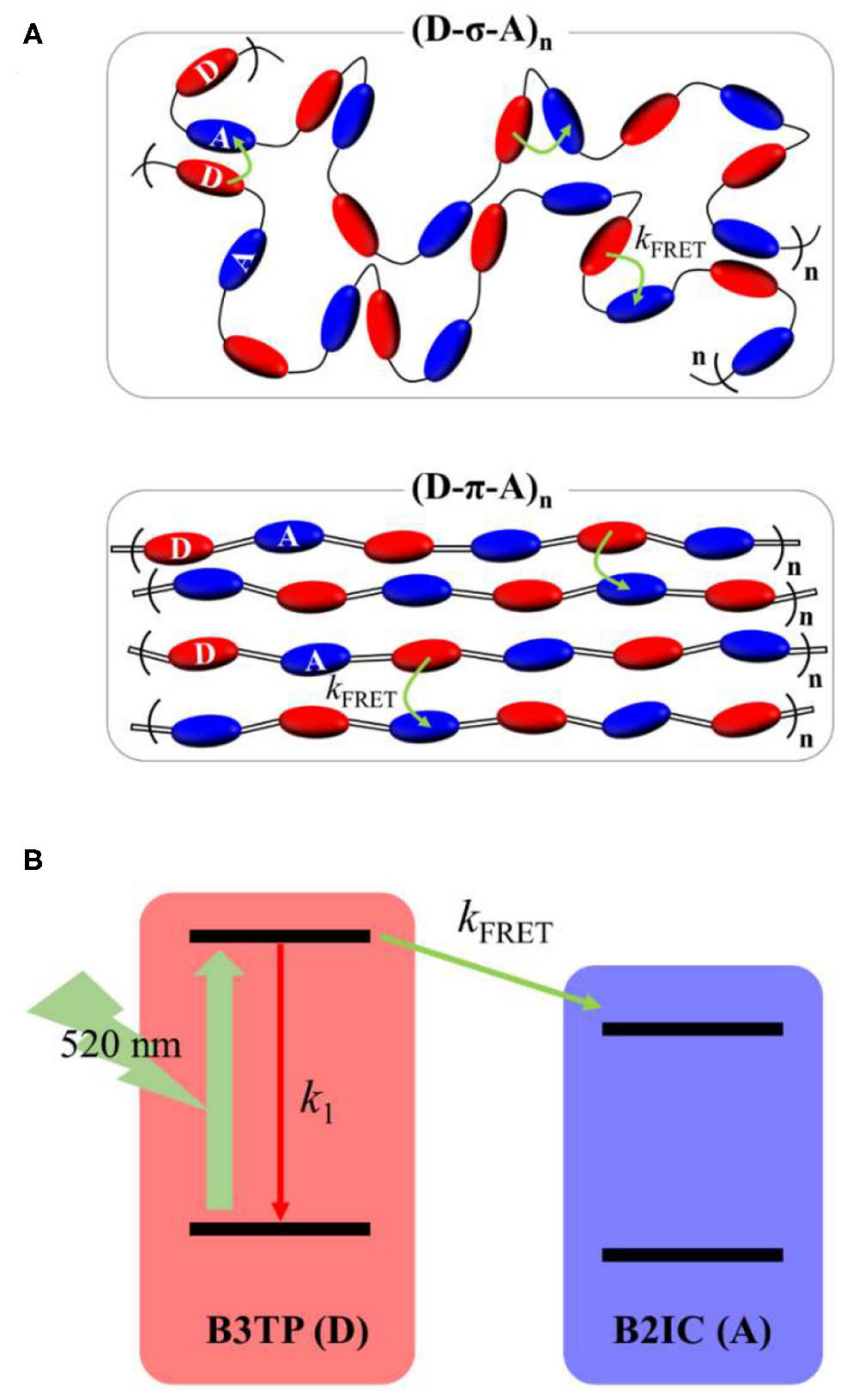
**(A)** Schematic illustration of **(D-σ-A)**_******n**_ and **(D-π-A)**_******n**_ in films and Förster resonance energy transfer (FRET) from the donor (**B3TP**, **D**) to acceptor (**B2IC**, **A**) units. Intramolecular and intermolecular (or interchain) FRET are indicated by curved arrows. **(B)** Kinetic model illustrating FRET in **(D-σ-A)**_******n**_ and **(D-π-A)**_******n**_.

To further understand the fluorescence quenching, the TRF signals of **B3TP**, **(D-σ-A)**_******n**_, and **(D-π-A)**_******n**_ in toluene were measured using the time-correlated single-photon counting method, as shown in Figure 3C. **B3TP**, **(D-σ-A)**_******n**_, and **(D-π-A)**_******n**_ in toluene were excited by 520 nm laser pulses and their TRF signals were measured at 590 nm to monitor the emission of the donor monomers (**B3TP**). The TRF signal of **(D-σ-A)**_******n**_ (Figure 3C) decayed faster than that of **B3TP** and **(D-π-A)**_******n**_ in toluene. The TRF signals were fitted to a multi-exponential function, and the average fluorescence lifetimes are summarized in Table 1. The average fluorescence lifetimes (τ_avg_ = 0.67 ns) of **B3TP** and **(D-π-A)**_******n**_ in toluene are the same within experimental error, even though the initial TRF decay of **(D-π-A)**_******n**_ is slightly faster than that of **B3TP**. The average fluorescence lifetime (τ_avg_ = 0.54 ns) of **(D-σ-A)**_******n**_ in toluene is slightly shorter than that of **B3TP** and **(D-π-A)**_******n**_. The difference in the TRF signals of **(D-σ-A)**_******n**_ and **(D-π-A)**_******n**_ in toluene results from their structural difference, caused by the linkers between the donor and acceptor units, as illustrated in Figure 3D. In **(D-π-A)**_******n**_, the π-conjugated linker (*sp*^2^) between the donor and acceptor units is rigid, which fixes the donor and acceptor far from each other, as shown in Figure 3D. Therefore, FRET between the donor and acceptor units in **(D-π-A)**_******n**_ is less likely. In contrast, in **(D-σ-A)**_******n**_, the *sp*^3^ alkyl chain between the donor and acceptor units is flexible; thus, a folded structure can feasibly be formed as shown in Figure 2C. Thus, intramolecular FRET between the donor and acceptor units in the folded sections of **(D-σ-A)**_******n**_ is likely, as schematically illustrated in Figure 3D. The optical properties of **B3TP**, **B2IC**, **(D-σ-A)**_******n**_, and **(D-π-A)**_******n**_ in the films were further studied, as shown in Figure 4. The UV-visible absorption spectra of **B3TP**, **B2IC**, **(D-σ-A)**_******n**_, and **(D-π-A)**_******n**_ in the films were slightly red-shifted relative to those in toluene (Supplementary Figure 6). The spectral features indicate that the donor and acceptor units are closely packed and intermingled in the films, as illustrated in Figure 5A. This structural arrangement in the films leads to a significant change in the fluorescence properties of **(D-σ-A)**_******n**_ and **(D-π-A)**_******n**_. The steady-state emission spectra of **(D-σ-A)**_******n**_ and **(D-π-A)**_******n**_ in Figure 4B show the emission peak of the acceptor monomers (**B2IC**). This indicates completely efficient FRET from the large donor to acceptor units in **(D-σ-A)**_******n**_ and **(D-π-A)**_******n**_. In the films, the donor and acceptor units are closely located so that intermolecular FRET from the donor and acceptor units in **(D-σ-A)**_******n**_ and **(D-π-A)**_******n**_ becomes very efficient. Figure 4C shows the TRF signals of **B3TP**, **(D-σ-A)**_******n**_, and **(D-π-A)**_******n**_ in the films. The TRF signal of **B3TP** in the films is found to decay faster than that of **B3TP** in toluene because of the “aggregation causing quenching” effect. The TRF signal of **(D-π-A)**_******n**_ in the films decays much faster than that of **(D-π-A)**_******n**_ in toluene, resulting from additional intermolecular (or interchain) FRET between the π-extended donor and acceptor units. The TRF signals of the films were fitted by a multi-exponential function, and the average fluorescence lifetimes are summarized in Table 1.

**Table 1:** Exponential fit of time-resolved fluorescence signals, $S\left(t\right)=\underset{i}{\sum }A_{i}\exp\left(-t/\tau_{i}\right)$, of **B3TP**, **(D-σ-A)**_******n**_, and **(D-π-A)**_******n**_ in toluene and films.

	**B3TP**		**(D-σ-A)**_***********n*******_		**(D-π-A)**_***********n*******_	
	**Toluene**	**Film**	**Toluene**	**Film**	**Toluene**	**Film**
*A*_1_	0.94	0.77	0.46	0.98	0.30	0.97
τ_1_	0.64 ns	0.16 ns	0.17 ns	0.10 ns	0.32 ns	0.11 ns
*A*_2_	0.06	0.23	0.52	0.02	0.69	0.03
τ_2_	1.1 ns	0.54 ns	0.79 ns	0.64 ns	0.79 ns	0.70 ns
*A*_3_	–	–	0.02	–	0.01	–
τ_3_	–	–	2.4 ns	–	2.8 ns	–
τ_avg_	0.67 ns	0.25 ns	0.54 ns	0.11 ns	0.67 ns	0.13 ns

*The average lifetime was calculated as $\tau_{avg}=\;\underset{i}{\sum }A_{i}\tau_{i}/\underset{i}{\sum }A_{i}$*.

By using a simple kinetics model (Figure 5B), the intramolecular and intermolecular (or interchain) FRET rate constants can be estimated (Supplementary Table 1). As shown in Table 1, the average fluorescence lifetime of **(D-σ-A)**_******n**_ (τ_avg_ = 0.54 ns) in toluene is shorter than that of **B3TP** (τ_avg_ = 0.67 ns) in toluene because of the intramolecular FRET from the donor units (**B3TP**, **D**) to the acceptor units (**B2IC**, **A**). The intramolecular FRET rate constant of **(D-σ-A)**_******n**_ in toluene was estimated to be *k*_FRET_ = 0.36 ns^−1^. In the same way, the FRET rate constants of **(D-σ-A)**_******n**_ and **(D-π-A)**_******n**_ in the films were determined to be *k*_FRET_ = 5.09 and 3.69 ns^−1^, respectively. The FRET rate constant was found to be much greater in the films than in toluene. This is because the donor and acceptor units in **(D-σ-A)**_******n**_ and **(D-π-A)**_******n**_ are much more closely located in the films, and intramolecular and intermolecular FRET are both very efficient in the films, as illustrated in Figure 5A.

## Conclusions

To study the role of linkers in energy transfer, we designed and synthesized two dyad polymer systems. First, the π-extended donor monomers (**B3TP**, **D**) with an A-D-A structure and the π-extended acceptor monomers (**B2IC**, **A**) with an A-D1-D2-D1-A structure were synthesized. These donor and acceptor units were linked together by two different linkers (non-conjugated alkyl and π-conjugated linker) to synthesize two types of D-A repeating polymers, **(D-σ-A)**_******n**_ and **(D-π-A)**_******n**_.

The photophysical properties of **(D-σ-A)**_******n**_ and **(D-π-A)**_******n**_ were comprehensively investigated by UV-visible absorption, steady-state emission, and TRF spectroscopy, in conjunction with DFT calculations. DFT calculations show that the π-extended donor and acceptor monomers (**B3TP** and **B2IC**) are planar; **D-σ-A** and **D-π-A** have similar energy profiles, originating from the donor and acceptor units, and exhibit intramolecular charge transfer characteristics upon electronic excitation. A folded structure is possible for **(D-σ-A)**_******n**_ because of the flexible non-conjugated alkyl linkers. **(D-σ-A)**_******n**_ and **(D-π-A)**_******n**_ exhibit similar UV-visible absorption properties. Their fluorescence was found to be quenched due to FRET from the large donor to acceptor units in **(D-σ-A)**_******n**_ and **(D-π-A)**_******n**_. In toluene, FRET is more efficient for **(D-σ-A)**_******n**_ than for **(D-π-A)**_******n**_. It is likely that the donor and acceptor units are more closely located in the folded structure of **(D-σ-A)**_******n**_, which increases the speed of FRET. In the films, the fluorescence of **(D-σ-A)**_******n**_ is more significantly quenched than that of **(D-π-A)**_******n**_, and intramolecular and intermolecular (or interchain) FRET occur more efficiently. The FRET rate constants were determined using a simple kinetics model.

For the two conjugated polymers bearing π-extended donor and acceptor monomers, the linkers between the donor and acceptor units are shown to play an important role in determining the overall structure of the polymers, thereby influencing the optical properties. The flexible alkyl linkers in the repeating group of the polymers lead to more structural freedom in solution, while the rigid π-conjugated linkers provide less flexibility to the fully conjugated polymers. This structural flexibility can significantly impact the optical properties, and can be tailored depending on the application of the donor- and acceptor-based conjugated polymers for a wide range of research fields.

## Data Availability Statement

The original contributions presented in the study are included in the article/Supplementary Materials, further inquiries can be directed to the corresponding author/s.

## Author Contributions

DC and SP conceived the research. Chemical synthesis was carried out by NK, SHP, and MC. Spectroscopic experiments and DFT calculations were done by YK. YK and SP analyzed the spectroscopic data. NK, YK, DC, and SP wrote the manuscript. All authors contributed to the article and approved the submitted version.

## Conflict of Interest

The authors declare that the research was conducted in the absence of any commercial or financial relationships that could be construed as a potential conflict of interest.
