# Supplementary material for: Dynamics of Photoinduced Energy Transfer in Fully and Partially Conjugated Polymers Bearing π-Extended Donor and Acceptor Monomers
Source: Front Chem. 2020 Nov 5;8:605403. doi: 10.3389/fchem.2020.605403 (PMC7674937; doi:10.3389/fchem.2020.605403)
Supplement: Supplementary file 1 [file Data_Sheet_1.PDF]

Supplementary Material

Dynamics of Photoinduced Energy Transfer in Fully and Partially Conjugated Polymers Bearing π-Extended Donor and Acceptor Monomers

Youngseo Kim†, Na Yeon Kwon†, Su Hong Park, Min Ju Cho, Dong Hoon Choi*, and Sungnam Park*

Department of Chemistry, Research Institute for Natural Sciences, Korea University, 145 Anam-ro, Seongbuk-gu, Seoul 02841, Korea

Corresponding Authors:
dhchoi8803@korea.ac.kr
Fax: +82-2-3290-3121, Tel: +82-2-3290-3140.
spark8@korea.ac.kr
Fax: +82-2-3290-3121, Tel: +82-2-3290-3144.

†These authors contributed equally to this work.

1) Synthesis of compounds

The chemicals required to synthesize the polymer used in this study were purchased from Sigma-Aldrich, Acros Organics, and Tokyo Chemical Industry and were used without further purification. Compounds **1**, **2**, and **3** were synthesized according to published methods. (Lin et al., 2016;Cho et al., 2017)

Compound M1, B2IC: M1 was synthesized via Knoevenagel condensation of compound **1** (0.50 g, 0.204 mmol) and compound **2** (0.120 g, 0.448 mmol) with pyridine as a catalyst. The mixture was heated at 70 °C for 6 h. The resulting product was purified by silica gel column chromatography with only dichloromethane to yield 0.600 g (98%) of **M1** as a dark blue solid. ^{1}H NMR (500 MHz, $CDCl_3$): δ (ppm) 8.89 (s, 2H), 8.81 (s, 1H), 8.54 (d, J = 9.7 Hz, 1H), 7.85 (d, J = 7.5 Hz, 2H), 7.74 (m, 4H), 7.68 (s, 2H), 7.65 (s, 2H), 7.42 (s, 2H), 7.29 (m, 2H), 7.23 (s, 2H), 7.09–7.17 (m, 32H), 6.92 (s, 2H), 2.89 (s, 4H), 2.58 (t, 16H), 1.72 (m, 2H), 1.60 (m, 16H), 1.29–1.36 (m, 64H), 0.87–0.92 (t, 36H). MALDI-TOF: m/z 2955.82 [M^+]. Elemental Anal. Calcd. for ($C_{188}H_{192}Br_2N_4O_2S_8$): C, 76.39; H, 6.55; N, 1.90; S, 8.68. Found: C, 76.68; H, 6.48; N, 1.92; S, 8.52.

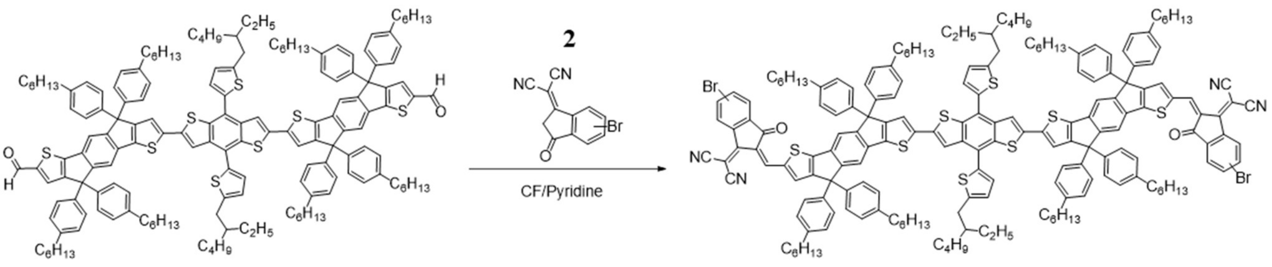

1 B2IC

Scheme S1. Synthetic procedure for **B2IC** (**M1**).

Compound B3TP: Compound **3** (100 mg, 0.06 mmol) was added to the mixture of 4-bromophenylacetonitrile (24.6 mg, 0.126 mmol) with solution of KO^{t}Bu in dry THF (20 mL). The reaction mixture was stirred for 4h. The mixture was poured into water, and extracted with $CHCl_3$. The organic layer was washed with water and then dried over Na_2SO_4. After concentration, the crude product was purified with column chromatography on silica gel with a mixture of $CHCl_3$ and hexane

(1:1) as eluant. (80 mg, 67%) as dark green solid. ^{1}H NMR (500 MHz, $CHCl_3$): δ (ppm) 7.61 (s, 2H), 7.55-7.48 (m, 10H), 7.44 (s, 2H), 7.32 (d, J = 3.3 Hz, 2H), 7.22 (d, J = 3.9 Hz, 2H), 7.11 (d, J = 4.6 Hz, 2H), 6.95 (d, J = 3.5 Hz, 2H), 2.92-2.90 (t, 4H), 2.83-2.79 (t, 4H), 2.78-2.75 (t, 4H), 1.70-1.67 (m, 8H), 1.47-1.28 (m, 56H), 1.01-0.98 (m, 8H), 0.96-0.94 (m, 8H), 0.89-0.86 (m, 14H). MALDI-TOF: m/z 1932.34 [M^+]. Elemental Anal. Calcd. for ($C_{108}H_{126}Br_2N_2S_{10}$): C, 67.12; H, 6.57; N, 1.45; S, 16.59. Found: C, 67.23; H, 6.46; N, 1.41; S, 16.62.

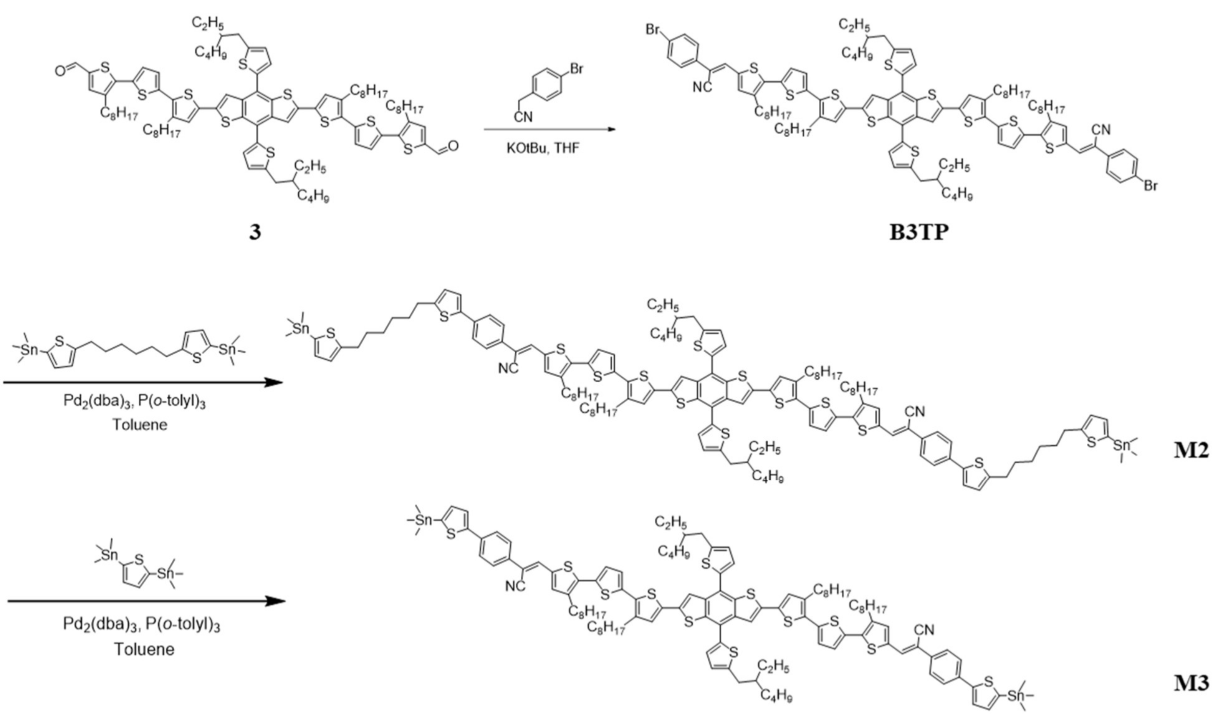

Scheme S2. Synthetic procedure for **B3TP**, **M2**, and **M3**

Compound M2: **B3TP** (100 mg, 0.051 mmol) and 1,6-bis(5-(trimethylstannyl)thiophen-2-yl)hexane (298 mg, 0.51 mmol) in toluene (20 mL) was degassed twice with N_2 gas followed by the addition of $Pd_2(dba)_3$ (2.3 mg, 5 mol%) and P(o-tolyl)$_3$ (3.1 mg, 10 mol%). After being stirred at 100 °C for 24 h, the reaction mixture was poured into cold acetonitrile. The precipitate was filtered and wash to afford compound M2 (150 mg, 41%) as a dark green solid. ^{1}H NMR (500 MHz, $CHCl_3$): δ (ppm) 7.58 (d, J = 2.7 Hz, 2H), 7.55 (s, 6H), 7.47 (s, 2H), 7.43 (s, 2H), 7.39 (s, 2H), 7.32 (d, J = 3.3 Hz, 2H), 7.18 (d, J = 3.7 Hz, 2H), 7.15 (d, J = 3.3 Hz, 2H), 7.06 (d, J = 3.6 Hz, 2H), 7.02 (d, J = 3.0 Hz, 2H), 6.96 (d, J = 3.6 Hz, 2H), 6.95 (d, J = 3.0 Hz, 2H), 2.93-2.92 (m, 4H), 2.88-2.85 (t, 4H), 2.82-2.72 (m, 20), 1.70-

1.68 (m, 28H), 1.47-1.28 (m, 60H), 1.04-0.95 (m, 20H), 0.90-0.87 (m, 8H), 0.34 (m, 18H). Elemental Anal. Calcd. for ($C_{142}H_{176}N_2S_{14}Sn_2$): C, 65.67; H, 6.83; N, 1.08; S, 17.28. Found: C, 65.43; H, 6.95; N, 1.10; S, 17.32.

Compound M3: B3TP (120 mg, 0.062 mmol) and 2,5-bis(trimethylstannyl)thiophene (250 mg, 0.62 mmol) in toluene (20 mL) was degassed twice with N_2 gas followed by the addition of $Pd_2(dab)_3$ (2.8 mg, 5 mol%) and $P(o\text{-tolyl})_3$ (3.7 mg, 10 mol%). Synthetic set up and reaction conditions were the same as described of M2 synthetic procedure. Compound M3 (90 mg, 64%) as a dark green. ^1H NMR (500 MHz, $CHCl_3$): δ (ppm) 7.65-7.60 (m, 10H), 7.53 (s, 2H), 7.47-7.44 (m, 6H), 7.33 (d, J = 3.3 Hz, 2H), 7.21 (d, J = 3.6 Hz, 2H), 7.18 (d, J = 3.3 Hz, 2H), 7.10-7.09 (m, 4H), 6.95 (d, J = 3.3 Hz, 2H), 2.93-2.76 (m, 16H), 1.69 (m, 28H), 1.47-1.28 (m, 60H), 1.01-0.94 (m, 22H), 0.89-0.83 (m, 8H), 0.41 (m, 18H). Elemental Anal. Calcd. for ($C_{122}H_{148}N_2S_{12}Sn_2$): C, 64.70; H, 6.59; N, 1.24; S, 16.99. Found: C, 64.65; H, 6.45; N, 1.22; S, 16.82.

2) Experiments

The molecular weight characteristics (M_n and PDI) of the polymers were determined relative to polystyrene (PS) standards by gel permeation chromatography (GPC) using 1,2-dichlorobenzene (*o*-DCB). The masses of the synthesized compounds were determined by matrix-assisted laser desorption ionization time of fight (MALDI-TOF) mass spectrometry (MALDI-TOF/TOF™ 5800 system /AB SCIEX) at the Korea Basic Science Institute (Seoul).

Spectroscopic measurements: UV-visible absorption spectra of the sample solutions were measured in a quartz cuvette with a 10 mm path length by using a Cary 100 spectrometer (Varian). Steady-state emission spectra were measured using a HITACHI F-7000 fluorescence spectrometer. Time-resolved fluorescence (TRF) signals, $S(t)$, were collected at a series of wavelengths by using a time-correlated single-photon counting (TCSPC) technique.(Joung et al., 2017) The samples were excited by a 520 nm pulse (LDH-P-C-520, PicoQuant). The emitted fluorescence was measured by using a photomultiplier

tube (PMA 182, PicoQuant). The instrumental response function (IRF) of our TCSPC setup was measured to be about 130 ps in FWHM. TRF signals were fitted by a multi-exponential function, $S(t) = \sum_i A_i \exp(-t/\tau_i)$. The average lifetime was determined by $\tau_{\text{avg}} = \sum_i A_i\tau_i \,/\, \sum_i A_i$.

3) Quantum chemical calculations

The optimized structures, frontier orbitals, electronic absorption and emission spectrum, and natural transition orbitals (NTOs) of **B3TP**, **B2IC**, **D-σ-A**, and **D-π-A** were obtained using the density functional theory (DFT) and time-dependent DFT methods with the B3LYP functional and 6-31G(d) basis set that is implemented in the Gaussian 16 package. In addition, the folded structure of **D-σ-A** is optimized using the DFT methods with B3LYP-d3 functional and 6-31G(d) basis set.

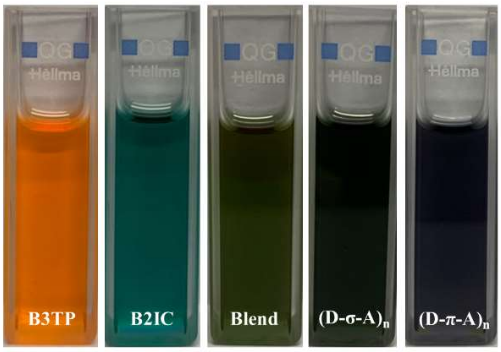

Figure S1. Photographs of the macromonomers, blend, and polymers in toluene.

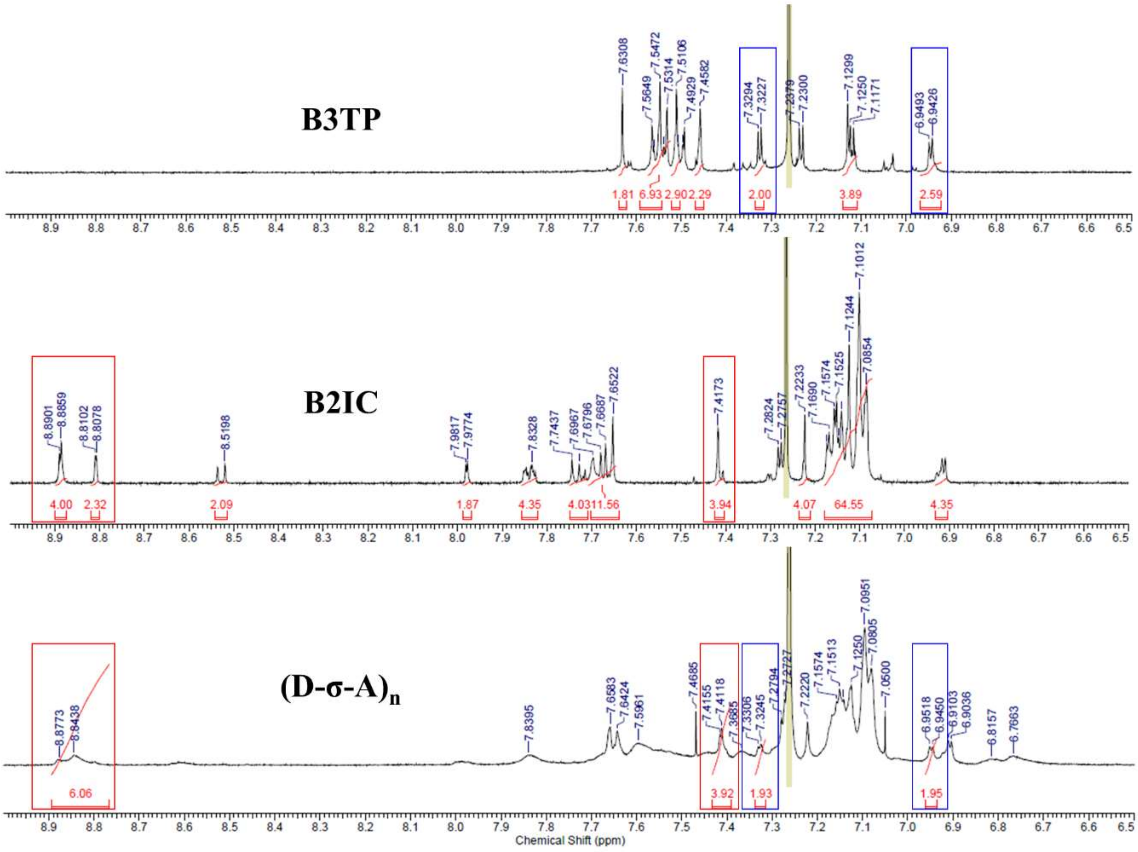

Figure S2. ^{1}H NMR spectra of **B3TP**, **B2IC**, and **(D-σ-A)$_n$**.

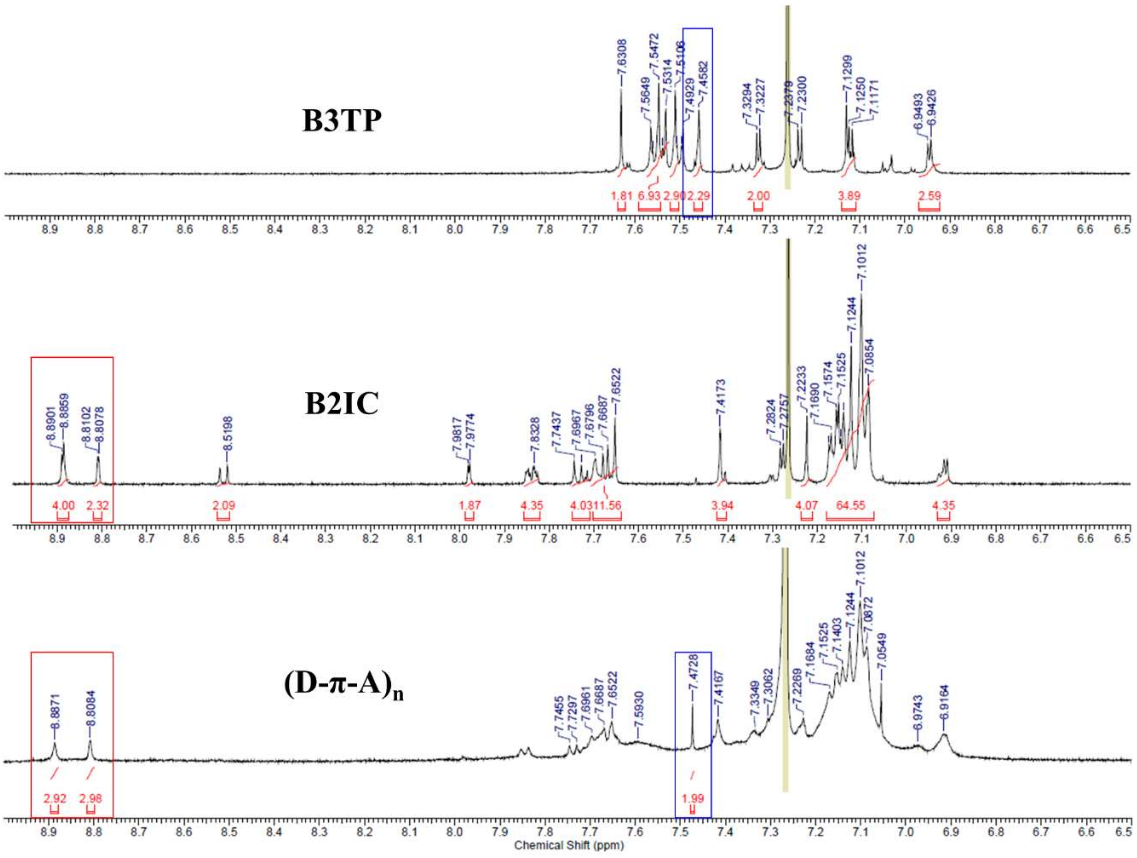

Figure S3. ^{1}H NMR spectra of **B3TP**, **B2IC**, and **(D-π-A)$_{\mathbf{n}}$**.

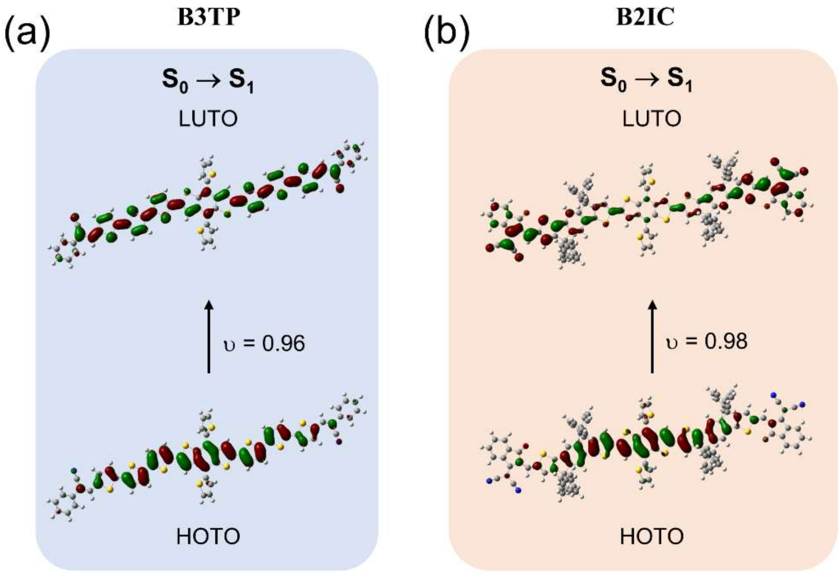

Figure S4. Natural transition orbitals (NTOs) for $S_0 \rightarrow S_1$ transition of (a) **B3TP** and (b) **B2IC**.

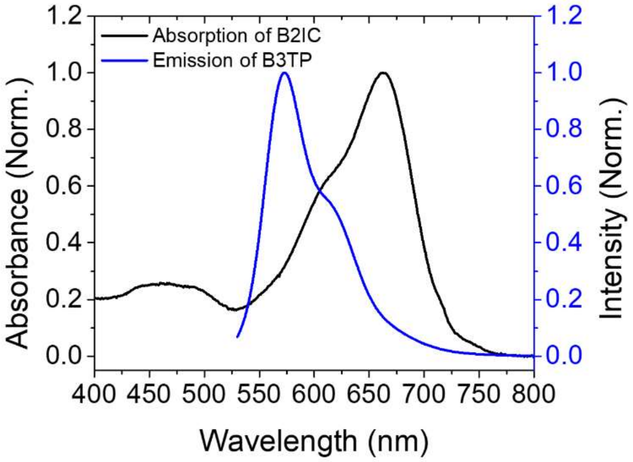

Figure S**5.** UV-visible absorption spectrum of **B2IC** and emission spectrum of **B3TP** in toluene.

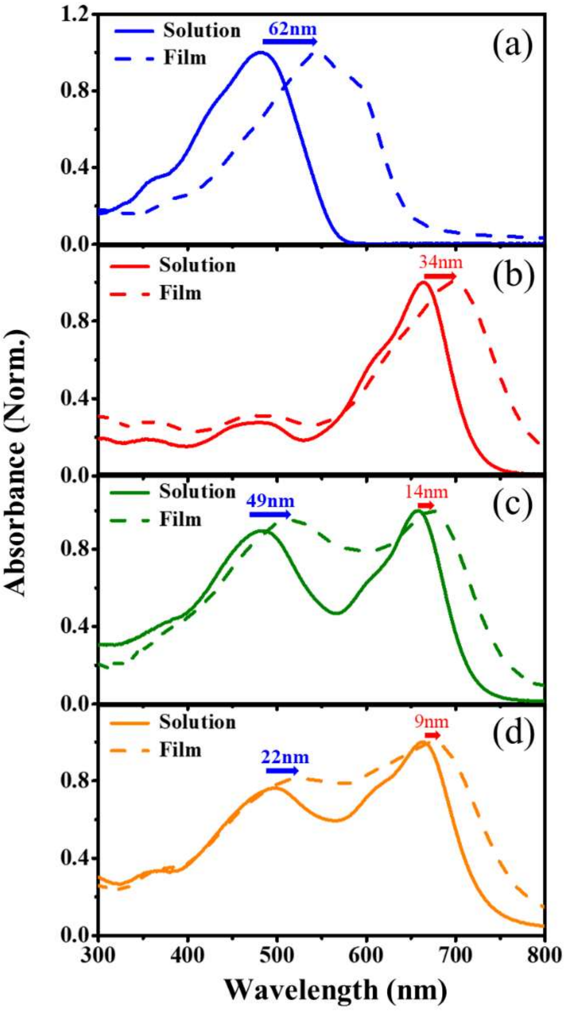

Figure S6. UV-visible absorption spectra of (a) **B3TP**, (b) **B2IC**, (c) **(D-σ-A)$_n$**, and (d) **(D-π-A)$_n$** in toluene and film.

Table S1. Kinetic parameters of **B3TP**, **(D-σ-A)$_n$**, and **(D-π-A)$_n$** in toluene and films.

	**B3TP**		**(D-σ-A)$_n$**		**(D-π-A)$_n$**	
	Toluene	Film	Toluene	Film	Toluene	Film
k_1	1.49 ns^{-1}	4.00 ns^{-1}	1.49 ns^{-1}	4.00 ns^{-1}	1.49 ns^{-1}	4.00 ns^{-1}
k_{FRET}	-	-	0.36 ns^{-1}	5.09 ns^{-1}	-	3.69 ns^{-1}

Kinetic parameters of **B3TP**, **(D-σ-A)$_n$**, and **(D-π-A)$_n$** are obtained by using following equations, $\tau_{\mathbf{B3TP}} = 1/k_1$, $\tau_{(\mathbf{D-\sigma-A})_\mathbf{n}} = 1/(k_1 + k_{\mathrm{FRET}})$, and $\tau_{(\mathbf{D-\pi-A})_\mathbf{n}} = 1/(k_1 + k_{\mathrm{FRET}})$, respectively.

Table S2. Electrochemical properties of **B3TP**, **B2IC**, **(D-σ-A)$_n$**, and **(D-π-A)$_n$**.

	E_g^{opt} [a] (eV)	E_{ox}^{onset} [b] (V)	E_{red}^{onset} [b] (V)	Energy (eV) [c]	
				HOMO	LUMO
**B3TP**	1.91	0.83	-	-5.26	-3.35
**B2IC**	1.58	1.13	-0.43	-5.56	-4.00
**(D-σ-A)$_n$**	1.63	1.04	-0.48	-5.47	-3.95
**(D-π-A)$_n$**	1.60	1.05	-0.48	-5.48	-3.95

[a] Calculated from the absorption edge of the films: $E_g^{opt} = 1240/l_{cut\ off}$. [b] The values were obtained from cyclic voltammograms. *Sample: film on Pt electrode. The optical bandgaps were obtained from absorption spectra of film samples. [c] HOMO(eV) = LUMO(eV) – E_g^{opt}(eV), E_{ox}^{onset} of ferrocene = 0.37 eV, Calculated according to the equation: $E_{HOMO/LUMO} = -e(4.8 + E_{ox/red} - E_{ferrocence})$ (eV)

References

Cho, M.J., Park, G.E., Park, S.Y., Kim, Y.-U., and Choi, D.H. (2017). Structural optimization of large acceptor–donor–acceptor-type molecules for improved performance of fullerene-free polymer solar cells. *RSC Advances* 7**,** 38773-38779.

Joung, J.F., Kim, S., and Park, S. (2017). Ionic effects on the proton transfer mechanism in aqueous solutions. *Phys. Chem. Chem. Phys.* 19**,** 25509-25517.

Lin, Y., Li, T., Zhao, F., Han, L., Wang, Z., Wu, Y., He, Q., Wang, J., Huo, L., Sun, Y., Wang, C., Ma, W., and Zhan, X. (2016). Structure Evolution of Oligomer Fused-Ring Electron Acceptors toward High Efficiency of As-Cast Polymer Solar Cells. *Advanced Energy Materials* 6.
